# Pim-1 kinase is a target of miR-486-5p and eukaryotic translation initiation factor 4E, and plays a critical role in lung cancer

**DOI:** 10.1186/1476-4598-13-240

**Published:** 2014-10-24

**Authors:** Wenshuai Pang, Xin Tian, Fan Bai, Ruiyu Han, Juan Wang, Haitao Shen, Xianghong Zhang, Yueping Liu, Xia Yan, Feng Jiang, Lingxiao Xing

**Affiliations:** Department of Pathology, Hebei Medical University, Shijiazhuang, Hebei China; Department of Pathology, Second Hospital of Hebei Medical University, Shijiazhuang, Hebei China; Department of Pathology, Tumor Hospital of Hebei Medical University, Shijiazhuang, Hebei China; Department of Pathology, University of Maryland School of Medicine, Baltimore, Maryland USA

## Abstract

**Background:**

Pim-1 kinase is a proto-oncogene and its dysregulation contributes to tumorigenesis and progression of a variety of malignancies. Pim-1 was suggested as a therapeutic target of cancers. The functional relevance of Pim-1 and the mechanism underlying its dysregulation in lung tumorigenesis remained unclear. This study aimed to investigate if Pim-1 has important functions in non-small-cell lung cancer (NSCLC) by: 1) evaluating the clinicopathologic significance of Pim-1 through analysing its expression in 101 human NSCLCs tissues using quantitative PCR, Western Blot and immunohistochemical studies, 2) determining its role in NSCLC and drug resistance using *in vitro* assays, and 3) investigating the regulatory mechanism of Pim-1 dysregulation in lung tumorigenesis.

**Results:**

Pim-1 was upregulated in 66.2% of the lung tumor tissues and its expression was significantly related to advanced stage (P = 0.019) and lymph node metastasis (P = 0.026). Reduced Pim-1 expression suppressed NSCLC cell growth, cell cycle progression and migration *in vitro*. Pim-1 was a novel target of miR-486-5p determined by luciferase report assay, and ectopic miR-486-5p expression in cancer cells reduced Pim-1 expression. Furthermore, eukaryotic translation initiation factor 4E (eIF4E) controlled the synthesis of Pim-1 in NSCLC cells, and its expression was positively associated with that of Pim-1 in NSCLC tissue specimens (r = 0.504, p < 0.001). The downregulated miR-486-5p and upregulated eIF4E in NSCLC cells led to the overexpression of Pim-1 by relieving the inhibitory effect of the 3′-UTR or 5′-UTR of Pim-1 mRNA, respectively. Moreover, Pim-1 knockdown sensitized NSCLC cells to cisplatin and EGFR tyrosine kinase inhibitor, gefitinib.

**Conclusions:**

Pim-1 kinase could be a critical survival signaling factor in NSCLC, and regulated by miR-486-5p and eIF4E. Pim-1 kinase may provide a potential target for diagnosis and treatment for lung cancer.

**Electronic supplementary material:**

The online version of this article (doi:10.1186/1476-4598-13-240) contains supplementary material, which is available to authorized users.

## Background

Pim-1 belongs to the active serine/threonine kinase family. It functions as a proto-oncogene whose activation could promote the development of cancer in animal models [1, 2]. Several target proteins of Pim-1 are involved in apoptosis, cell cycle regulation, signal transduction pathways and transcriptional regulation, which are linked to cell survival. Thus, overexpression of Pim-1 in cancer cells can substantially contribute to malignant transformation, tumor progression and poor prognosis [3]. Elevated levels of Pim-1 have been found in human hematological malignancies [4] and certain solid cancers, including prostate cancer [5], head and neck squamous cell carcinomas [6], colon carcinoma [7], and pancreatic ductal adenocarcinoma [8]. Furthermore, targeting Pim-1 sensitized prostate and colon cancer cells to chemotherapeutic agents [7, 9, 10], and improved the efficacy of AKT inhibitors and epidermal growth factor receptor (EGFR) targeted therapies in prostate cancer [11, 12]. Therefore, targeting Pim-1 may provide a new therapeutic approach for the cancers.

Lung cancer is the leading cause of cancer death, mainly due to the lack of effective treatment and the development of resistance to chemotherapeutic agents. The development of effective therapeutics for lung cancer is urgently needed. Pim-1 expression at mRNA and protein level in non-small-cell lung cancer (NSCLC) had been reported in few study, however the results were controversial. Warnecke et al. [13] reported that Pim-1 expressions at mRNA and protein levels were significantly decreased in NSCLC tissues compared to normal lung tissues. However, Nasser et al. [14] indicated that Pim-1 expression at mRNA level was considerably elevated in NSCLC tissues compared to the paired normal lung tissues. Jin et al. [15] recently found that Pim-1 protein expression correlated with advanced clinical stage and lymph node metastasis of patients using immunohistochemical assays. Until now, the functions of Pim-1 in the development and progression as well as the mechanism underlying its dysregulation in tumorigenesis of NSCLC are still uncertain.

It has been well established that microRNAs (miRNAs) play important gene-regulatory roles by pairing to the 3′-UTR of specific target mRNAs [16]. MiRNAs can posttranscriptionally regulate the expression of hundreds of their target genes, thereby controlling a wide range of biological processes. Pim-1 has been found to be negatively regulated by some miRNAs, such as miR-15 [7], miR-1 [15], miR-328 [17], miR-33a [18] and miR-210 [19]. Previously, we have identified a set of 26 miRNAs whose abnormal expressions are associated with human NSCLC. Among the 26 miRNAs, miR-486-5p is one of the most downregulated miRNAs in lung tumor tissues. We have found that miR-486-5p is a valuable diagnostic biomarker for early detection of NSCLC in sputum and plasma [20–22]. We also demonstrated that miR-486-5p acted as a tumor-suppressor in the development and progression of NSCLC through targeting ARHGAP5 [23]. To further identify additional novel targets of miR-486-5p that may play an important function in the development and progression of NSCLC, we predict its targeted genes using bioinformatic assays. Interestingly, Pim-1 was the top 25 high-scoring candidates among the 32853 genes predicted by miRecords (http://mirecords.biolead.org/). This observation led us to explore the underlying mechanism of miR-486-5p on the expression of Pim-1 in NSCLC.

Eukaryotic translation initiation factor 4E (eIF4E), the component of eIF4F translation initiation complex, is the least abundant of the initiation factor. EIF4E is considered as the rate-limiting component for initiation of cap-dependent translation [24]. EIF4E overexpression can cause preferential translation of mRNAs containing excessive secondary structure in their 5′-UTR that are normally inefficiently translated, like growth promoting protein and oncogenic proteins MYC, CCND1 and VEGF. By this mechanism, eIF4E overexpression in cancer cells is associated with cancer-related events such as transformation, angiogenesis, invasion and metastasis [25]. Pim-1 has a 400-nucleotide long, 76% G/C-rich and highly structured 5′-UTR in its mRNA, which has the potential to inhibit translation under normal cellular conditions [26]. However, whether eIF4E is involved in the regulation of Pim-1 expression and tumorigenesis of NSCLC remained uncertain.

In the study, we aimed to evaluate the roles of Pim-1 kinase in tumorigenesis and drug resistance of NSCLC, and to explore the possible mechanism underlying Pim-1 dysregulation in lung carcinogenesis by: evaluating the clinicopathologic significance of Pim-1 through analysing the expression in 101 human NSCLCs tissues using quantitative PCR, Western Blot and immunohistochemical studies, determining its role in NSCLC and drug resistance using *in vitro* assays, and investigating the regulatory mechanism of miR-486-5p and eIF4E on Pim-1 expression in lung tumorigenesis.

## Results

### Pim-1 protein is frequently overexpressed in human NSCLC

**Figure 1:**
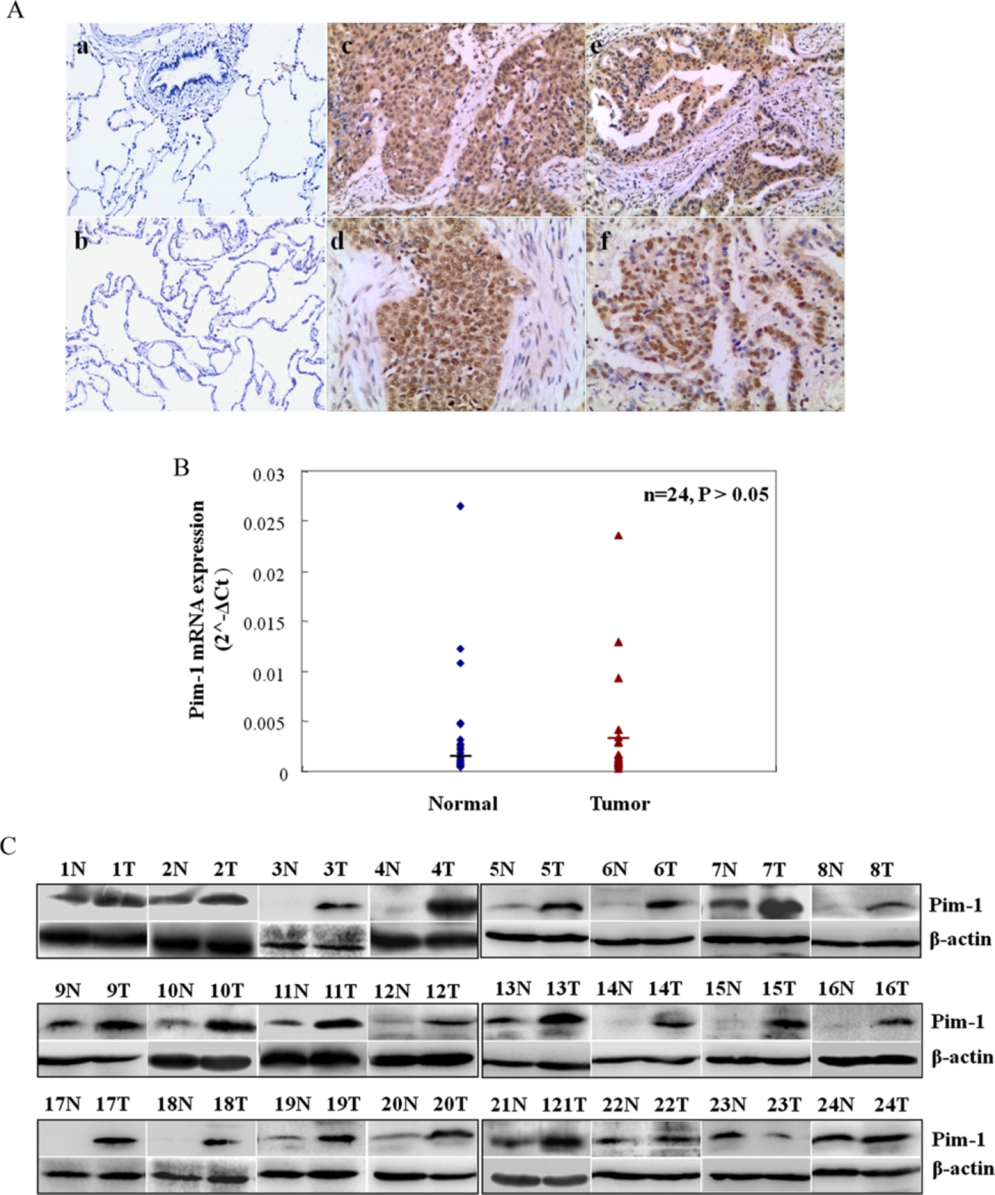
**Pim-1 protein is frequently overexpressed in human NSCLC. (A)** Pim-1 expression was detected by immunohistochemistry analysis with Pim-1 antibody in normal lung tissue (a-b), squamous cell carcinoma (c-d), and adenocarcinoma (e-f) of lung. (c and e): Pim-1 expression mainly seen in cytoplasm of carcinoma cells, SP, 20 × 10; (d and f): Pim-1 expression in nucleus of carcinoma cells prominently, SP, 40 × 10. **(B)** The level of Pim-1 mRNA in 24 cases of human NSCLC and matched normal lung tissues by qRT-PCR assay. **(C)** Pim-1 protein expression in tumor tissues (T) and paired normal lung tissues (N) from the same sets as in **(B)** by Western blot assay.

To evaluate clinical significance of Pim-1 expression, we performed immunohistochemical studies on formalin-fixed and paraffin-embedded (FFPE) sections of 77 NSCLC. Pim-1 staining was detected in the nucleus and cytoplasm of carcinoma cells (Figure 1A). Positive staining of Pim-1 was found in 66.2% (51/77) of NSCLC tissues (Table 1), but only 14.3% (3/21) in normal lung tissues. Furthermore, Pim-1 expression was significantly higher in NSCLC tissues than in adjacent normal tissues (P < 0.001). In addition, Pim-1 expression in NSCLC tissues was significantly associated with tumor size (P = 0.002), lymph node metastasis (P = 0.026), histological type (P < 0.001) and clinical staging (P = 0.019) in NSCLC patients (Table 1). Therefore, upregualtion of Pim-1 was associated with aggressive behaviour of lung cancer. Analysis of expression level of the gene may help predict outcome of NSCLC.

**Table 1:** **Clinicopathologic characteristics of the NSCLC patients with different stages and histologies by Pim-1 expression**

Characteristics	Number of patients	Pim-1 expression		P
		Positive	Negative	
**All cases**	**77**	**51**	**26**	
Age (y)				0.807
<=60	37	24	13	
>60	40	27	13	
**Gender**				
Male	53	37	16	0.324
Female	24	14	10	
**Histology**				<0.001
Squamous cell carcinoma	28	24	4	
Adenocarcinoma	44	27	17	
Other types	5	0	5	
**Tumor size**				0.002
<=3 cm	43	22	21	
>3 cm	34	29	5	
**Lymphnodemetastasis**				0.026
Negative	31	16	15	
Positive	46	35	11	
**Metastasis**				
No	51	28	23	0.003
Yes	26	23	3	
**Stage**				
I	23	13	10	0.019
II	18	11	7	
III	10	4	6	
IV	26	23	3	

NOTE: P values <0.05 were considered statistically significant.

We further compared Pim-1 expression in matched pairs of frozen tumor and normal tissues from 24 NSCLC patients by using qRT-PCR and Western Blot assays. At mRNA level, Pim-1 was upregulated only in 37.5% (9/24) tumor tissues when compared with the paired normal lung tissues. Furthermore, there was no significant difference in the average level of Pim-1 mRNA between tumor and paired normal lung tissues (Figure 1B, P > 0.05). In contrast, at protein level, Pim-1 expression was overexpressed in 87.5% (21 of 24) tumor tissues than in adjacent normal tissues (Figure 1C and Additional file 1: Table S1). Wilcoxon matched pair rank test further demonstrated that Pim-1 protein expression in tumors was statistically higher than that in normal tissues (Z = −4.257, P < 0.001). Collectively, Pim-1 protein is frequently overexpressed in NSCLC.

### Knockdown Pim-1 inhibits cell growth, cell cycle progression and migration of NSCLC cells *in vitro*

**Figure 2:**
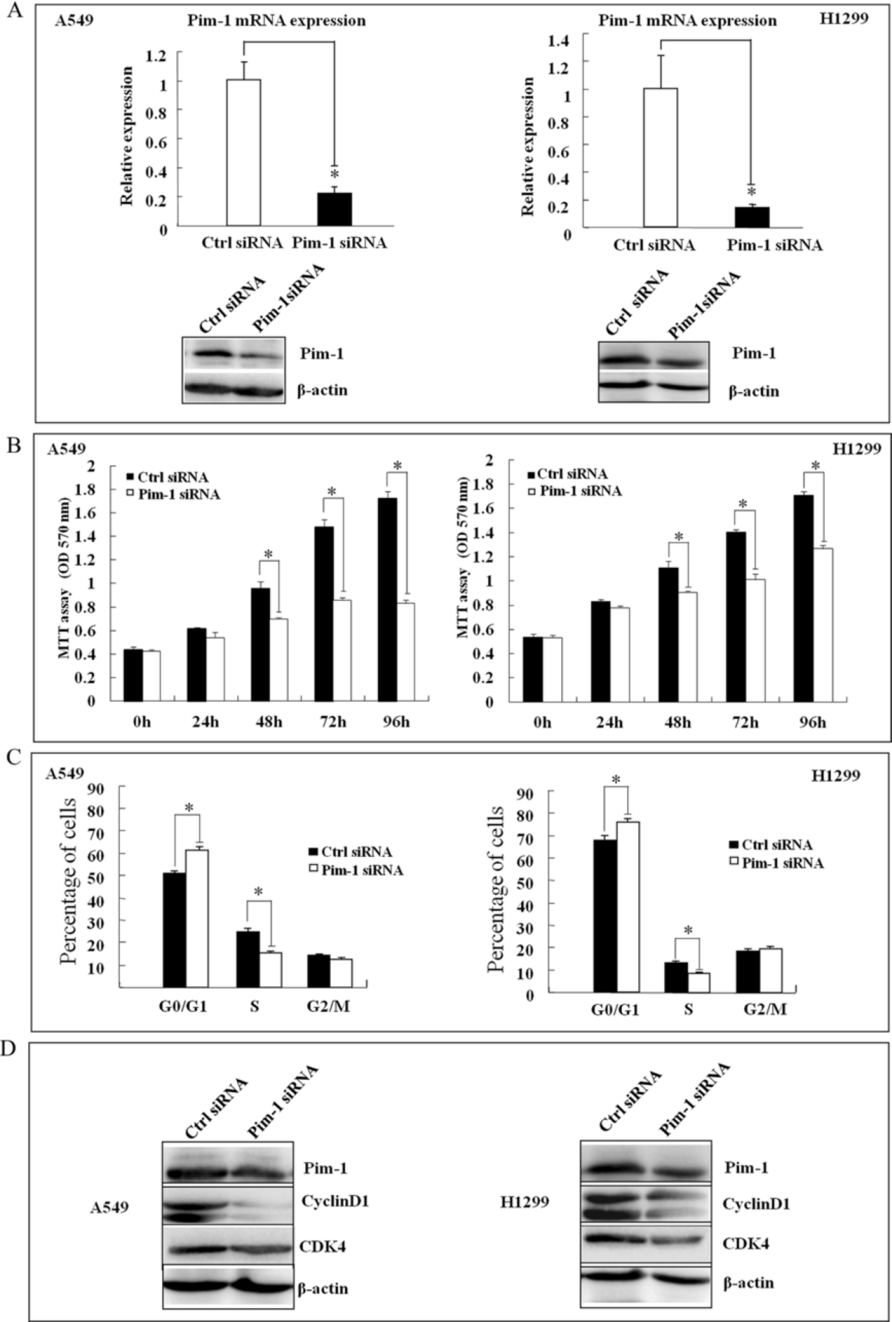
**Effects of Pim-1 on cell growth and cell cycle progression in NSCLC cells** ***in vitro*** **. (A)** A549 and H1299 cells were transfected with Pim-1 siRNA at a final concentration of 50nM. Pim-1 expression was detected using qRT-PCR (Top) and Western blot (Bottom) at 48 h posttransfection. **(B)** A549 and H1299 cells were transfected as in **(A)**. In the indicated time periods (0-96 h) posttransfection, cell viability was evaluated using MTT assay. **(C)** Cells were stained using propidium iodide (PI) 48 h posttransfection and the cell cycle distribution was analyzed by FACS. **(D)** CyclinD1 and CDK4 protein expressions were detected in A549 and H1299 cells after transfection with si-Pim-1 by Western Blot assay. All data were obtained from three independent experiments and shown as mean ± SD and *P <0.05.

**Figure 3:**
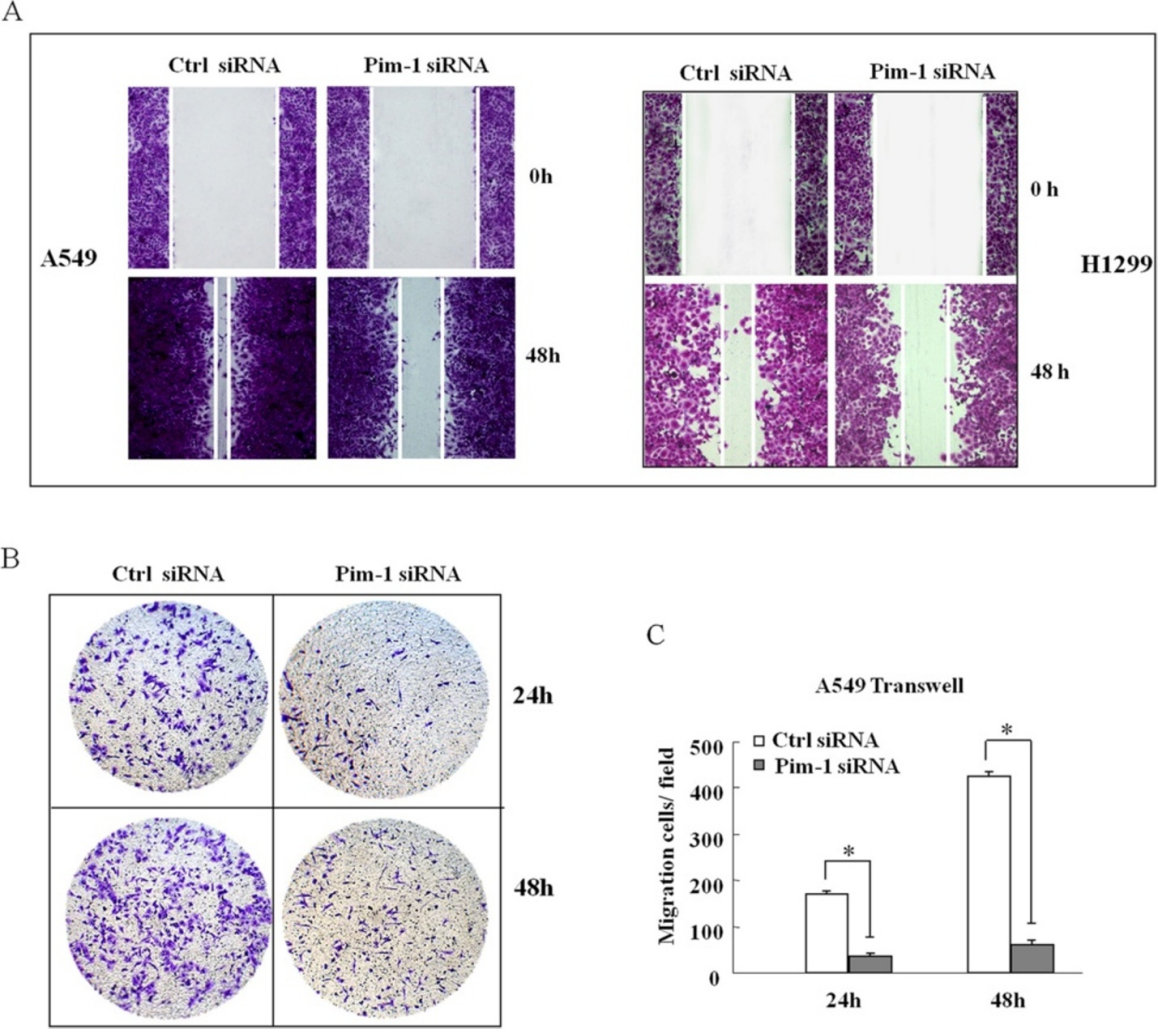
**Effects of Pim-1 on migration of NSCLC cells** ***in vitro*** **. (A)** A549 and H1299 cells were transfected with Pim-1 siRNA at a final concentration of 50nM. The ability of cell motility was evaluated in A549 and H1299 cells after transfection with si-Pim-1 for 48 h using wound scratch assay. A uniform scratch was made in each confluent layer culture, the extent of wound closure was monitored under microscope, and photographs were taken at 0 and 48 h. **(B)** Knockdown of Pim-1 reduces A549 cell migration in the “Transwell assay”. Cells that migrated to the bottom chamber containing serum-supplemented medium were stained with crystal violet, visualized under microscope, and photographed at 24 h and 48 h, respectively. **(C)** Average number of cells migrated to the bottom chamber in five fields was counted manually. Data are shown as mean ± SD. Similar results were obtained in three independent experiments. *P < 0.05.

To explore the functions of Pim-1 in lung tumorigenesis, we used specific siRNA against Pim-1 (si-Pim-1) to reduce the expression of Pim-1 in NSCLC cells. Successful knockdown of Pim-1 was confirmed by qRT-PCR and Western Blot assays (Figure 2A). Cell growth was significantly suppressed in cells transfected with si-Pim-1 compared with the control cells at 48 h, 72 h and 96 h after transfection by MTT assay (P < 0.05, Figure 2B). The antiproliferative effect of the siRNA-mediated Pim-1 knockdown was also confirmed in clone formation assay in A549 cells (Additional file 2: Figure S1). Furthermore, Flow Cytometry (FCM) results indicated that the proportion of cells in G_0_/G_1_ phase was increased significantly, whereas cells in S phase was significantly decreased in si-Pim-1 transfected cells compared to the control (P <0.05, Figure 2C). Cyclin D1 and CDK4 proteins (the key proteins in controlling the cell cycle transition from G_1_ to S phase) were assessed by Western Blot. As shown in Figure 2D, the levels of Cyclin D1 and CDK4 protein were considerably decreased in si-Pim-1 transfected cells, further indicating that Pim-1 downregulation led cells to be arrested at G_0_/G_1_ phase.Monolayer wound healing assay was performed to observe the role of si-Pim-1 transfection in A549 and H1299 cells. As shown in Figure 3A, the speed which cells migrated towards the scratch was lower in si-Pim-1 transfected cells when compared with the control ones. Transwell assay in A549 cells further indicated that loss of Pim-1 considerably suppressed the migratory ability of the NSCLC cells (Figure 3B and C). Taken together, the observations suggest that Pim-1 might have oncogenic functions in NSCLC.

### Pim-1 expression is negatively regulated by miR-486-5p at posttranscriptional level in NSCLC

**Figure 4:**
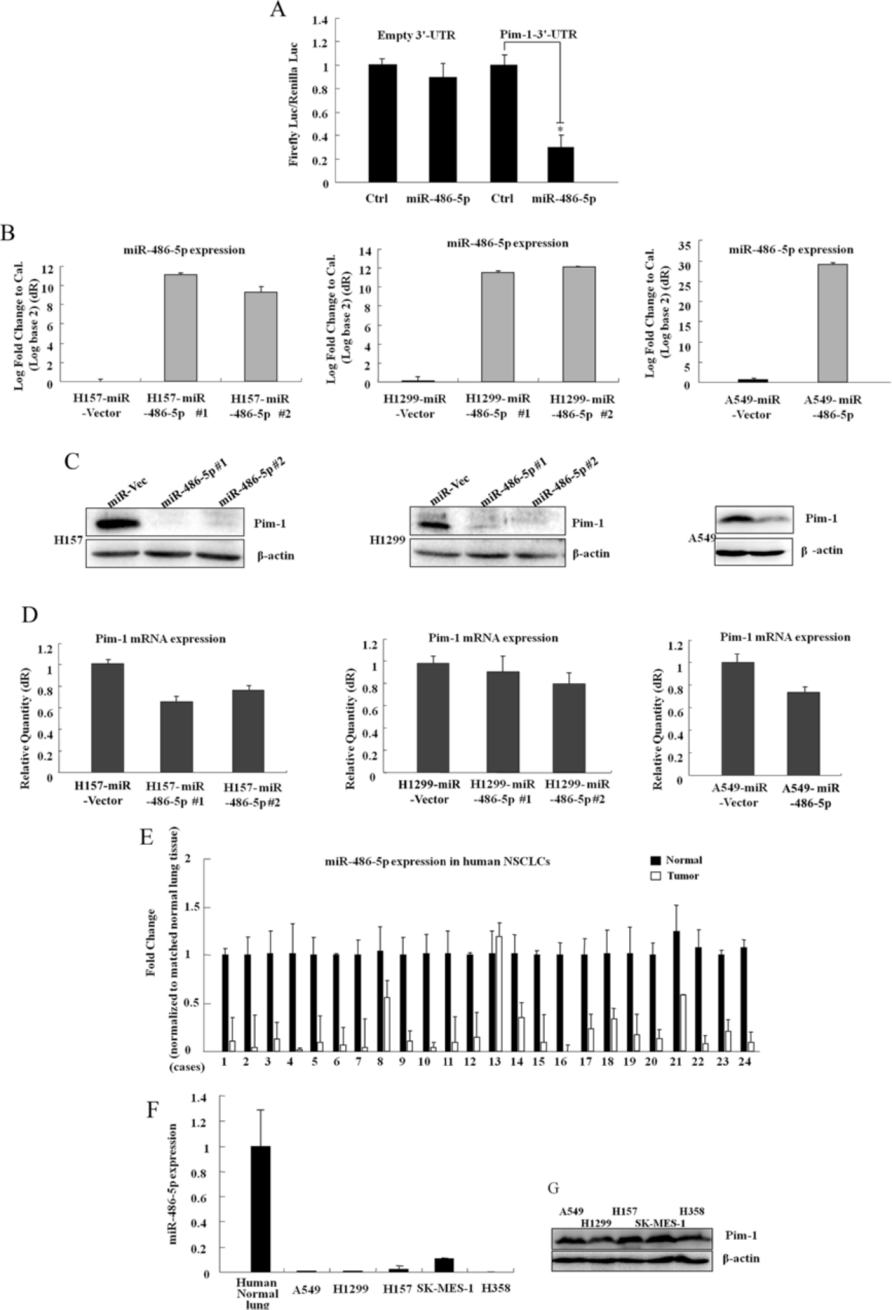
**MiR-486-5p regulates Pim-1 expression at posttranscriptional level. (A)** Luciferase activity controlled by Pim-1-3′-UTR was inhibited by miR-486-5p. A549 cells were co-transfected with firefly luciferase-3′-UTR (pGL3 or pGL3-Pim-1-3′UTR) and pRL-TK vector (as internal control) along with miR-486-5p or control miR. After 48 h, firefly luciferase and Renilla luciferase were measured. **(B)** Stable cell lines expressing miR-486-5p or the vector were selected with puromycin and used for the following experiment. **(C)** Pim-1 protein was determined in A549,H1299 and H157 cells stable expressing miR-486-5p by Western Blot assay. **(D)** The level of Pim-1 mRNA was determined in A549,H1299 and H157 cells stable expressing miR-486-5p by qRT-PCR assay. **(E)** miR-486-5p is frequently down-regulated in tumor tissue than the matched normal tissue from the same 24 cases of human primary NSCLC as mentioned in Figure 1C. **(F)** miR-486-5p expressions were detected in NSCLC cell lines A549, H1299, H157, SK-MES-1 and H358 using real-time qRT-PCR. **(G)** Pim-1 protein expressions in the five NSCLC cell lines were detected by Western Blot assay.

We have previously shown that miR-486-5p may act as a tumor suppressor whose downregulation could contribute to progression and metastasis of NSCLC [23]. To further elucidate the mechanisms responsible for the tumor-suppressive abilities of miR-486-5p, we use bioinformatic analysis to identify additional novel targets of miR-486-5p. Interestingly, miR-486-5p was the top ten miRNAs that bind to the 3′-UTR region of Pim-1 (miRecords: http://mirecords.biolead.org/).To determine whether Pim-1 could be regulated by miR-486-5p, we performed luciferase reporter assay. The luciferase activity of Pim-1-3′-UTR was reduced by ~70% in cells expressing miR-486-5p compared with those expressing the control (Figure 4A). Furthermore, we generated stable H1299, H157 and A549 cells overexpressing miR-486-5p, and measured Pim-1 expression using Western blot assay. Forced expression of miR-486-5p in the cells resulted in a decrease of Pim-1 protein expression compared with control cells (Figure 4B, C). However, this was not accompanied by a significant reduction in mRNA expression in cells overexpressing miR-486-5p relative to their control cells, respectively (Figure 4D). Together, miR-486-5p might directly bind to the 3′-UTR sequences of Pim-1, as well as inhibiting its expression mainly through posttranscriptional regulation.

We further analyzed miR-486-5p expression in the same panel of frozen NSCLC tissues that were used for the analysis of Pim-1 expression. MiR-486-5p was downregulated in 87.5% (21of 24) tumor tissues compared with the matched normal lung tissues (Figure 4E and Additional file 1: Table S1), whereas Pim-1 protein was upregulated in 87.5% (21 of 24) tumor tissues by Western bolt analysis (Figure 1C). The level of miR-486-5p in tumors was significantly lower than that in normal tissues using Wilcoxon matched pair rank test (Z = −3.971, P < 0.001), which was consistent with our previous report [20, 23]. In addition, in five NSCLC cell lines (A549, H1299, H157, SK-MES-1 and H358) that showed decreased miR-486-5p expression relative to that of human normal lung tissue (Figure 4F), Pim-1 protein expressions were all indicated at relatively higher level (Figure 4G and Additional file 3: Figure S2). Overall, the findings indicated that miR-486-5p negatively regulated Pim-1 expression at posttranscriptional level, and the downregulated miR-486-5p in NSCLC conferred to Pim-1 upregulation by relieving the inhibitory effect of the 3′-UTR.

### Pim-1 expression is controlled by eIF4E at translational level, and is positively associated with eIF4E expression in NSCLC cells

**Figure 5:**
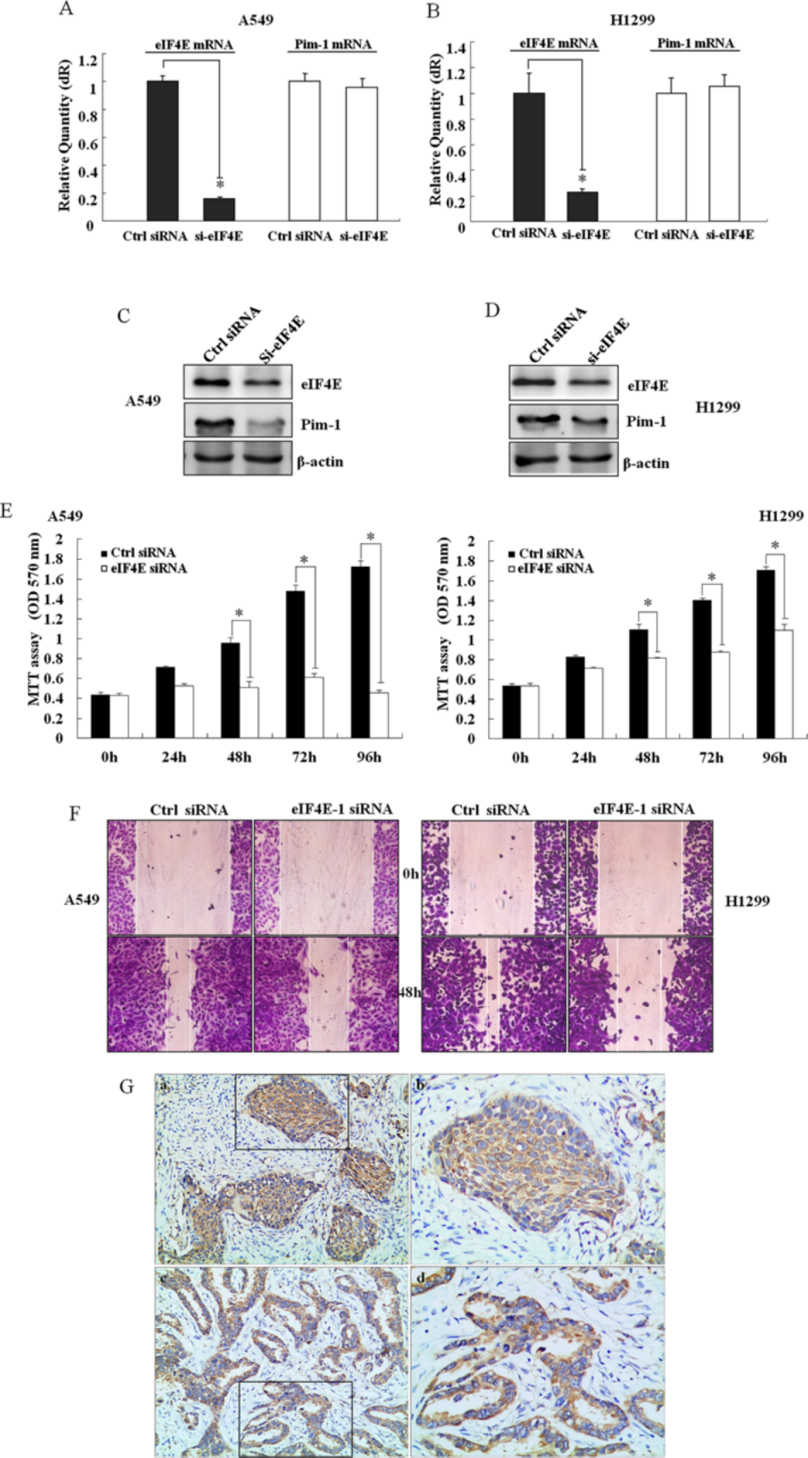
**Elevated eIF4E expression controls the synthesis of Pim-1 in NSCLC.** H1299 and A549 cells were transfected with eIF4E siRNA at a final concentration of 50nM. EIF4E and Pim-1 mRNAs were detected in H1299 **(A)** and A549 cells **(B)** using qRT-PCR 48 h posttransfection. Pim-1 and eIF4E protein expression was detected in H1299 **(C)** and A549 cells **(D)** using Western Blot assay 48 h psottransfection. **(E)** Cell growth was evaluated in A549 and H1299 cells after transfection with si-eIF4E for 0-96 h using MTT assay. **(F)** The ability of cell motility was evaluated in A549 and H1299 cells after transfection with si-eIF4E for 48 h using wound scratch assay. A uniform scratch was made in each confluent layer culture, the extent of wound closure was monitored under microscope, and photographs were taken at 0 and 48 h. Data are shown as mean ± SD. Similar results were obtained in three independent experiments. *P < 0.05. **(G)** EIF4E expression is frequently elevated in squamous cell carcinoma (a: SP, 100×; b: a segment magnified at 200×) and adenocarcinoma of lung by immunohistochemistry analysis (c: SP, 100×; d: a segment magnified at 200×).

To explore the effects of eIF4E on the regulation of Pim-1 expression and tumorigenesis of NSCLC, we knocked down eIF4E expression by siRNA and determined its impact on Pim-1 expression. The basal levels of eIF4E protein were all abundant in NSCLC cell line H1299, A549, H157, H460 and H358. Knockdown of eIF4E was confirmed in A549 (Figure 5A and C) and H1299 (Figure 5B and D) cells by qRT-PCR and Western blot analysis. In comparison with control cells, reduced levels of Pim-1 protein were detected in si-eIF4E transfected A549 (Figure 5C) and H1299 cells (Figure 5D). However, there was no change on Pim-1 mRNA expression after si-eIF4E transfection in both of the cells (Figure 5A and B). The results suggest that eIF4E expression controls the synthesis of Pim-1 protein in NSCLC cells. In addition, we found that silencing of eIF4E inhibited cell growth (Figure 5E) and the migratory ability of the NSCLC cells (Figure 5F). The observation is consistent with the roles of Pim-1 in NSCLC cells.

We further examined eIF4E expression and determined the relationship between eIF4E and Pim-1 expression in 69 human NSCLC specimens. Positive staining for eIF4E was found in the cytoplasm of the tumor cells (Figure 5G). EIF4E staining was found in 56 of 69 NSCLCs (81.1%), occurred 37 of 41 adenocarcinoma (90.2%), 16 of 23 squamous cell carcinoma (69.6%) and 3 of 5 other types (60.0%). As shown in Table 2, eIF4E expression was significantly associated with tumor diameter (P = 0.036). However, there were no significant associations between eIF4E expression and other important clinicopathological features, such as histological type, lymph node metastasis, and clinical staging in NSCLC patients (all P > 0.05, Table 2). Moreover, the correlation between the expressions of eIF4E and Pim-1 was explored using the Spearman’s rank test. 62.3% (43/69) of tumors positive on eIF4E were also Pim-1 positive, and 15.9% (11/69) eIF4E negative tumors were Pim-1 negative (Table 3). There was a positive correlation between eIF4E and Pim-1 expressions in the specimens (r = 0.504, P < 0.001). Therefore, eIF4E expression is positively associated with Pim-1, and elevated eIF4E expression could contribute to Pim-1 protein expression in NSCLC.

**Table 2:** **Clinicopathologic characteristics of the NSCLC patients with different stages and histologies by eIF4E expression**

Characteristics	Number of patients	eIF4E expression		P
		Positive	Negative	
**All cases**	69	56	13	
**Age (y)**				0.630
<=60	36	30	6	
>60	33	26	7	
**Gender**				
Male	47	36	11	0.200
Female	22	20	2	
**Histology**				0.058
Squamous cell carcinoma	23	16	7	
Adenocarcinoma	41	37	4	
Other types	5	3	2	
**Tumor size**				0.036
<=3 cm	35	25	10	
>3 cm	34	31	3	
**Lymphnodemetastasis**				0.338
Negative	29	22	7	
Positive	40	34	6	
**Metastasis**				
No	44	35	9	0.893
Yes	25	21	4	
**Stage**				
I	20	15	5	0.629
II	16	14	2	
III-IV	33	27	6	

NOTE: P values <0.05 were considered statistically significant.

**Table 3:** **Relativity of protein expressions of eIF4E and Pim-1 in NSCLC**

Pim-1 expression (n)	eIF4E expression (n)		Total
	Positive	Negative	
**Positive**	43	2	45
**Negative**	13	11	24
**Total**	56	13	69

(The association between the expression of eIF4E and Pim-1 in NSCLC was statistically significant (r = 0.504, p = 0.000).

### Pim-1 Knockdown increases the sensitivity of NSCLC cells to gefitinib and cisplatin

**Figure 6:**
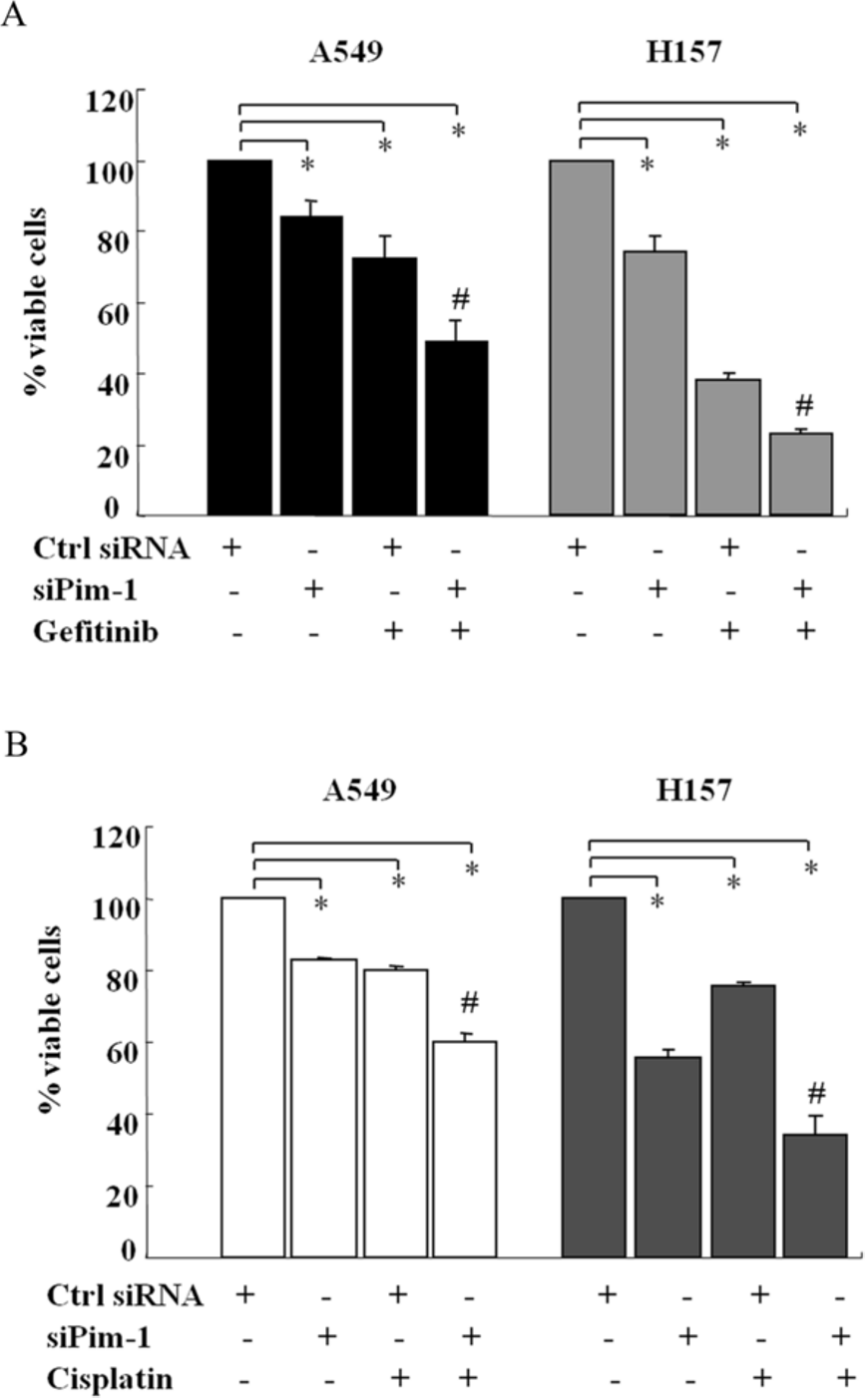
**Pim-1 Knockdown increased the sensitivity of NSCLC cells to gefitinib and cisplatin.** A549 and H157 cells were transfected with negative control or Pim-1 siRNA for 24 h and then exposed to 10 μM Gefitinib for 24 h **(A)**, 20 μM cisplatin for 48 h **(B)**, respectively. After the aforementioned treatments, cell viability was assessed by MTT assays. The data are means ± SDs of four replicate determinations. All data were obtained from three independent experiments and shown as mean ± s.d. and *P < 0.05 compared with each treatment alone.

Because Pim-1 has a critical role in NSCLC cell survival, we hypothesize that knockdown Pim-1 expression can sensitize NSCLC cells to therapeutic agents. To validate the hypothesis, cell survival rates were detected in A549, H157 and H1299 NSCLC cells transfected with control siRNA or si-Pim-1 combined with drug treatment using MTT assays. In A549 cells, the viability of cells was decreased significantly upon si-Pim-1(~16%, P = 0.031) or gefitinib (~28%, P = 0.002) treatment alone compared to the control cells. More importantly, silencing Pim-1 in combination with gefitinib reduced the viability of A549 cells by about 50% (P = 0.0001, Figure 6A). Similar synergistic effects were also obtained in combination of si-Pim-1 with gefitinib both in H157 (~80%) and H1299 cells (~60%) compared with their control cells (Figure 6A and Additional file 4: Figure S3A). The data would suggest that Pim-1 inhibition could improve the effect of gefitinib clinically. Moreover, Pim-1 knockdown caused increased susceptibility to cisplatin in A549, H157 and H1299 cells when compared with the control cells (Figure 6B and Additional file 4: Figure S3B). Collectively, loss of Pim-1 sensitized NSCLC cells to the chemotherapeutic drugs.

## Discussion

We found that Pim-1 protein was frequently upregulated in lung tumor tissues, and its expression was closely related to advanced stage and lymph node metastasis of NSCLC. Furthermore, reduced Pim-1 expression suppressed NSCLC cell proliferation, cell cycle progression and migration *in vitro*. In addition, Pim-1 expression was negatively regulated by miR-486-5p at posttranscriptional level, whereas positively regulated by eIF4E at translational level. Moreover, siRNA-mediated Pim-1 knockdown significantly increased the efficacy of EGFR tyrosine kinase inhibitors (EGFR-TKI) gefitinib and cispatin in NSCLC cells. Therefore, Pim-1 is a critical survival protein contributed to tumorgenesis and progression of lung cancer.

Pim-1 belongs to a family of naturally active serine/threonine kinases. Its activity is dependent on the amount of protein present in a given cell [27]. In this study, immunohistochemical results showed that Pim-1 expression was significantly elevated in human NSCLCs, and was closely related to lymph node metastasis and clinical stage of patients. Our observation was in agreement with the observation by Jin et al. using immunohistochemical method [15]. Furthermore, our western blot results demonstrated that increased Pim-1 protein level exited in lung tumors and NSCLC cell lines. However, the level of Pim-1 mRNA did not show significant difference between tumor and normal tissues. The discrepancies in mRNA and protein expression levels of the gene suggested that Pim-1 expression might be highly controlled by some mechanisms at posttranscriptional level. Indeed, we demonstrated that Pim-1 was regulated by miRNA and eukaryotic translation initiation factor 4E at posttranscriptional and translational level, respectively.

Data from ours [20, 21, 23] and others [28, 29] have indicated that reduced miR-486-5p expression is one of the most frequent molecular events in NSCLC. Therefore identifying and characterizing molecular targets of miR-486-5p will help deep understanding mechanisms underlying the development and progression of NSCLC. In the present study, we demonstrated that Pim-1 might be a novel target of miR-486-5p. Our study suggests a novel functionality of miR-486-5p in the context of Pim-1 inhibition at posttranscriptional level. Downregulation of miR-486-5p could contribute to Pim-1 upregulation, and hence promote the development and progression of NSCLC.

Availability of the eIF4E factor is especially important for mRNAs with long and structured 5′UTRs. These include, in particular, short-lived cell cycle regulators and oncoproteins, like c-MYC, Cyclin D1, BCL2 and MCL1 [24]. Cancer cells require continuous expression of these proteins, which may provide a therapeutic window for inhibitors of cap dependent translation in cancer. Li et al. recently reported that elevated eIF4E expression promoted proliferation and invasion of NSCLC cells, and contributed to development of acquired resistance to EGFR-TKIs [30]. It was reported that Pim-1 kinase is regulated by eIF4E at the translational level in NIH-3 T3 cells [26]. However, the relationship between eIF4E and Pim-1 in NSCLC has not been explored. In this present study, we demonstrated that eIF4E expression was elevated in NSCLCs, and was positively associated with that of Pim-1 in lung tumor specimens. Furthermore, inhibition of eIF4E expression could decrease Pim-1 protein expression in NSCLC cells. Therefore, Pim-1 expression might be regulated by miR-486-5p negatively at posttranscriptional level, whereas by eIF4E positively at translational level. Furthermore, the downregulated miR-486-5p and upregulated eIF4E in lung tumor could lead to the overexpression of Pim-1 protein by relieving the inhibitory effects of the 3′-UTR or 5′-UTR of Pim-1 mRNA, respectively.

Our *in vitro* experiments demonstrated that knockdown of Pim-1 could induce G_0_/G_1_ phase arrest of NSCLC cells. The induced G_0_/G_1_ phase arrest was accompanied by the decreased expression of Cyclin D1 and CDK4 proteins, which are necessary for G_1_-S transition. Furthermore, decreased Pim-1 expression inhibited the cell growth significantly. However, Pim-1 knockdown did not affect the apoptosis of NSCLC cells in our study (data not shown). These observations supported that Pim-1 might be essential for cell growth mainly through regulating G_1_-S cell cycle progression. Taken together, Pim-1 could have oncogenic effects and play a key role in cell survival of NSCLC.

Pim-1 has been suggested as an attractive therapeutic target for different types of cancers. For instance, SGI-1776, a Pim-1 kinase inhibitor, has been tested in a pediatric preclinical testing program for leukemia [31]. This Pim-1 kinase inhibitor has the potential to improve the efficacy of radiotherapy in NSCLC cells [32] and sensitize prostate cancer cells to gefitinib [12]. Here we found that siRNA-mediated Pim-1 knockdown increased the sensitivity of NSCLC cells to cisplatin. Furthermore, EGFR signaling was recently suggested to couple to activation of cap-dependent translation in EGFR wild-type NSCLC cells [33]. Resistance to EGFR-TKI can be mediated through multiple signaling pathways converging upon cap-dependent translation in EGFR-wild type NSCLC. Using an antisense oligonucleotide against eIF4E to disrupt cap-dependent translation can enhance sensitivity to erlotinib [33]. Interestingly, our present study showed that Pim-1 was involved in the efficacy of EGFR targeted therapies in NSCLC cells with wild-type EGFR. Therefore, as a cap-dependent translation protein, Pim-1 mediated drug resistance may relate to the elevated level of eIF4E in these cells. Future investigation of the molecular mechanisms of Pim-1 mediated drug resistance would help develop novel Pim-1-based therapeutic agents to improve the treatment of NSCLC.

## Conclusions

Pim-1 kinase could be a critical survival signaling factor in NSCLC, and regulated by miR-486-5p and eIF4E. Pim-1 kinase may provide a potential target for diagnosis and treatment for lung cancer.

## Methods

### Patients and clinical specimens

The study protocol was approved by the Institutional Review Boards of Tumor Hospital of Hebei Medical University. FFPE sections of lung tumor of 77 NSCLC patients were collected. Clinical characteristics of the patients are shown in Table 1. The 77 NSCLC patients consist of 24 females and 53 males, ages 27 to 72 years (median, 59 years). Forty-four patients were diagnosed with adenocarcinoma, 28 with squamous cell carcinoma, and 5 with other subtypes of NSCLC. Twenty-three patients had stage I disease, 18 patients had stage II disease, 10 patients had stage III disease, and 26 patients had stage IV. Furthermore, the frozen surgical tumor and corresponding normal lung tissues of 24 patients with NSCLC were also obtained. In each patient (#1 ~ #24), the tumor tissues and the normal lung tissues were collected from the same patient and the diagnosis of each frozen tissues was confirmed by hematoxylin-eosin staining. Among the 24 patients, 16 were diagnosed with adenocarcinoma and 8 with squamous cell carcinoma. Twelve patients had stage I and II disease, 12 patients had stage III and IV disease. All variants, including age, sex, stage, and lymph node metastasis, were obtained from clinical and pathologic records. None of the patients had received preoperative adjuvant chemotherapy or radiotherapy.

### Cell culture

A549, H1299, H358, H157 and SK-MES-1 human lung cancer cell lines were obtained from Cell Resource Center of Peking union Medical College and Cell Bank of Chinese Academy of Sciences. A549, H1299, H358 and H157 cells were maintained in RPMI-1640 medium containing 10% fetal bovine serum and penicillin/streptomycin. SK-MES-1 cells were maintained in MEM medium supplemented with 10% fetal bovine serum and penicillin/streptomycin. Cultures were maintained in a 5% CO_2_ humidified atmosphere at 37°C.

### Reagents and antibodies

Gefitinib was from Selleck Chemicals (Houston, TX, USA). Cisplatin was from Sigma-Aldrich Corporation. Antibodies specific to Pim-1 was from Epitomics (Hangzhou, China). Antibodies specific to Cyclin D1, CDK4 and eIF4E were from Cell Signaling Technology (Danvers, MA). Anti-β-actin was from Santa Cruz Biotechnologies (Santa Cruz, CA, USA). Pim-1 siRNA and eIF4E siRNA for *in vitro* experiment was from Genephma (Shanghai, China). MiR-486-5p expressing vector and the negative control vector were from Genecopoeia Co. (Guangzhou, China).

### RNA extraction, reverse transcription, and real-time RT-PCR

Total RNA from frozen tissues and cultured cells was extracted using TRIzol reagent (Life Technologies Corporation, Carlsbad, CA, USA). To detect Pim-1 and eIF4E mRNA expression, first-strand cDNA was first synthesized from 1 μg RNA using SuperScript II reverse transcriptase (Invitrogen), followed by PCR amplification using 5% cDNA for each reaction. The sequences of the primers were as follows: Pim-1 forward, 5′-TCATTAGATGGTGCTTGGCCCTGA-3′; Pim-1 reverse, 5′-TGTGGA GGTGGATCTCAGCAGTTT-3′; eIF4E forward, 5′-CAGAGACGAAGTGACC TCAATC-3′; eIF4E reverse, 5′-CATTAACAACAGCGCCACATAC-3′; β-actin forward, 5′-AGCGAGCATCCCCCAAAGTT-3′; β-actin reverse, 5′-GGGCACGA AGGCTCATCATT-3′. β-actin was used as an internal control. For miR-486-5p expression, cDNA was synthesized from total RNA with specific stem-loop primers and the TaqMan MicroRNA Reverse Transcription Kit (Applied Biosystems; Foster City, CA). Expression of miRNAs was analyzed by real-time PCR using the TaqMan MicroRNA Assay kit (Applied Biosystems; Foster City, CA) as described in our previous study [23]. U6 small nuclear RNA was used as an internal control. Experiments were repeated at least three times.

### siRNA silencing of Pim-1 or eIF4E

Transfections were performed using Lipofectamine 2000 reagent (Invitrogen, Grand Island, NY) following the manufacturer’s protocol. Cells were transfected with 50 nM Pim-1 siRNA (Genephma, Shanghai, China) or their relative control sequences, and the cells were harvested 48 hours after transfection. Sequence of siRNA targeting Pim-1 is 5′-CCUUCGAAGAAAUCCAGAATT-3′. Sequence of siRNA targeting eIF4E is 5′-GGACGAUGGCUAAUUACAU-3′. Sequence of negative control siRNA is 5′-UUCUCCGAACGUGUCACGU-3′. At least three independent experiments were carried out.

### Stably enforcing expression of miR-486-5p in NSCLC cells

To force expression of miR-486-5p in NSCLC cells, cells were transfected with miR-486-5p expressing vector (clone number: HmiR0130-MR04) or the empty vector (clone number: CmiR0001-MR04) (Genecopoeia Co., Guangzhou, China) by using Lipofectamine 2000 according to the manufacturer’s instructions. 48 h after transfection, cells were selected with 2 μg/ml puromycin to obtain the miR-486-5p expressing clones.

### Bioinformatics

The software programs miRecords (http://mirecords.biolead.org/) was used to predict the potential target genes of miR-486-5p.

### Pim-1 gene 3′ untranslated region (UTR) luciferase reporter assay

To create 3′-UTR luciferase reporter construct of Pim-1, 1200-bp sequences from putative miR-486-5p binding sites were synthesized and cloned into the pGL_3_-REPORT vector (Promega, Shanghai, China). The following primers were used to amplify the 3′-UTR of Pim-1: 5′- CCGACGCGTCAACATTTACAACTCATTCCA G-3′ and 5′-CCGCTCGAGTTTATTCAAAAAACGCCAAGT-3′. The amplified fragment was cloned into pGL_3_ luciferase report vector at Mlu I and Xho I sites. The sequence of plasmid (pGL_3_-Pim-1) was confirmed by DNA sequencing. Luciferase reporter assay was performed according to the previously described method [23, 34]. Briefly, cancer cells (5 × 10^4^ per well) were seeded in a 24-well plate the day before transfection, and then co-transfected with firefly luciferase-3′-UTR (pGL_3_ or pGL_3_-Pim-1, 500 ng) and pRL-TK vector (Promega) along with miR-486-5p mimics or control (Ribobio Co. Guangzhou, china). After two days, firefly luciferase and Renilla luciferase were measured by using synergy™ HT microplate reader (Biotek, Beijing, China) with the Dual-Glo® Luciferase assay system (Promega). Luciferase activities were normalized to Renilla luciferase activity. Experiments were repeated at least three times.

### MTT assay

Cells (4 × 10^3^ cells) were seeded onto 96-well plates in normal culture condition overnight followed by siRNA transfection specific for Pim-1 or eIF4E, and the cells viability was assessed in four replicates at 24, 48, 72 and 96 h after transfection. For drug treatment, cells with si-Pim-1 transfection were plated in 96-well plates for 24 h, and then gefitinib or cisplatin was added to the cultures. The cells viability was assessed at 24 or 48 h after the drug treatment. The experiments were performed at least three times.

### Colony formation assay

For colony formation assays, after 24-hour posttransfection, the cells were diluted and replated in six well plates. After ten days, visible colonies were fixed with methanol, stained with crystal violet, counted, and normalized to control group. The experiments were performed at least three times.

### Cell cycle analysis

Cells were collected and washed twice with cold PBS. For cell cycle analysis, cells were fixed in 70% ethanol at 4°C overnight. After centrifugating for 5 min at 1, 000 rpm at 4°C, the pellet was treated with 2 mg/ml RNase A at 37°C for 20 min and stained with 50 μg/ml propidium iodide (PI) containing 0.1% Triton X-100 and EDTA 0.02 mg/ml. Cell suspensions were analyzed by Flow Cytometry on a FACS Calibur system (BD Biosciences, Heidelberg, Germany). Cell cycle distribution were counted using Muticycle AV software. All measurements were performed in duplicate.

### Wound-healing assay

To determine cell migration, cells were seeded in 6-well plates and incubated to generate confluent cultures. Wounds were scratched in the cell monolayer using a 200 micropipette tip. The cells were rinsed with phosphate-buffered saline (PBS). The migration of the cells at the edge of the scratch was monitored at 48 h. The cells were stained and photographed. At least three independent experiments were carried out.

### Transwell assay

Cells were plated in medium without serum in the top chamber of a transwell (Corning, NY). The bottom chamber contained standard medium with 10% FBS. After incubation for 24 h or 48 h, the cells that had migrated to the lower surface of the membrane were fixed with formalin, stained with crystal violet, and photographed under microscope. Cell numbers were counted under a light microscope at X400 magnification. Experiments were carried out at least three times.

### Western blot

Total proteins (50-100 μg) extracted from cell lines or human NSCLC and adjacent normal tissues were analyzed by SDS-polyacrylamid gel electrophoresis and were transferred electrophoretically to nitrocellulose membrane. To evaluate expression of the proteins, blots were blocked with 5% nonfat milk in Tris-Buffered Saline and Tween 20, and incubated with a primary rabbit monoclonal antibody Pim-1, Cyclin D1, CDK4 or eIF4E. Antibody for β-actin (Santa Cruz Biotechnology, Santa Cruz, CA) was used as a control. The blots were then re-probed with secondary antibody and visualized by the chemiluminescence and scanned using ImageQuant LAS 4010 Imaging System (GE Healthcare Life Sciences, Piscataway, NJ).

### Immunohistochemistry assay

Immunohistochemistry staining was done on 77 FFPEs using rabbit monoclonal antibody against human Pim-1 (Epitomics). EIF4E expression was detected on 69 available FFPEs out of the 77 cases using rabbit monoclonal antibody against human eIF4E (Cell signaling Technology, Danvers, MA). All sections were examined and scored independently by two investigators without any knowledge of the clinicopathological data of the patients. The degree of immunostaining was evaluated by the proportion of positive staining tumor cells and the staining intensities. Scores representing the proportion of positively stained tumor cells were graded as: 0 (no positive tumor cells); 1 (<10%); 2 (10%–50%); and 3 (>50%). The intensity of staining was determined as: 0 (no staining); 1 (weak staining = light yellow); 2 (moderate staining = yellow brown); and 3 (strong staining = brown). The staining index (SI) was calculated as the product of staining intensity × percentage of positive tumor cells. SI score of greater than 1 was considered to be positive staining.

### Statistical analysis

Data are presented as mean ± SD. Statistical comparisons between experimental groups were analyzed by t test. Spearman’s correlation analysis was used to determine correlation between Pim-1, eIF4E expression level, and clinical characteristics of the NSCLC patients. Wilcoxon matched pair rank test was used to analyze Pim-1 and miR-486-5p expression level between tumor tissues and paired adjacent normal lung tissues. P < 0.05 was taken to indicate statistical significance.

## Electronic supplementary material

Additional file 1: Table S1: The relatively expression of miR-486-5p and Pim-1 protein in 24 cases of human primary NSCLC normalized to the paired normal lung tissues. (DOCX 16 KB)

Additional file 2: Figure S1: The effects of si-Pim-1 on cell proliferation in A549 cell in vitro. A549 cells were transfected with Pim-1 siRNA at a final concentration of 50nM. Cell proliferation was determined by clone formation assay. 500 cells were replated in six well plates after 24-hour posttransfection. After ten days culture, visible colonies were fixed with methanol, stained with crystal violet. The experiments were performed at least three times. (DOCX 674 KB)

Additional file 3: Figure S2: Pim-1 protein expressions were overexpressed in NSCLC cell lines (A549, H1299, H157, SK-MES-1 and H358) compared to the average level of Pim-1 in normal lung tissues by Western Blot assay. The level of Pim-1 protein in 24 cases of human normal lung tissue was used as negative control, and the relatively expression of Pim-1 protein in cell lines was normalized to the normal lung tissues. (DOCX 90 KB)

Additional file 4: Figure S3: Pim-1 Knockdown increased the sensitivity of H1299 cells to gefitinib and cisplatin. H1299 cells were transfected with negtive control or Pim-1 siRNA for 24 h and then exposed to 20 μM gefitinib for 24 h (A) or 20 μM cisplatin for 48 h (B) respectively. After the aforementioned treatments, cell viability was assessed by MTT assays. All data were obtained from three independent experiments and shown as mean ± s.d. #P < 0.05 compared with each treatment alone. (DOCX 59 KB)
