# Supplementary material for: Pim-1 kinase is a target of miR-486-5p and eukaryotic translation initiation factor 4E, and plays a critical role in lung cancer
Source: Mol Cancer. 2014 Oct 24;13:240. doi: 10.1186/1476-4598-13-240 (PMC4213487; doi:10.1186/1476-4598-13-240)
Supplement: Supplementary file 1 — Additional file 1: Table S1: The relatively expression of miR-486-5p and Pim-1 protein in 24 cases of human primary NSCLC normalized to the paired normal lung tissues. (DOCX 16 KB) [file 12943_2014_1440_MOESM1_ESM.docx]

# Additional files

**Additional file1 – Supplementary Table S1:**

**The relatively expression of miR-486-5p and Pim-1 protein in 24 cases of human primary NSCLC normalized to the paired normal lung tissues.**

| No. | miR-486(T/N) | Pim-1 protein(T/N) |
| --- | --- | --- |
| 1 | 0.103192 | 2.639183 |
| 2 | 0.041295 | 2.623915 |
| 3 | 0.123956 | 99.28834 |
| 4 | 0.01509 | 5.58162 |
| 5 | 0.086846 | 5.67623 |
| 6 | 0.066986 | 2.38443 |
| 7 | 0.042132 | 1.958502 |
| 8 | 0.560583 | 2.0881 |
| 9 | 0.099098 | 3.427865 |
| 10 | 0.045437 | 2.169345 |
| 11 | 0.074842 | 2.901871 |
| 12 | 0.139942 | 1.493065 |
| 13 | 1.168777 | 2.412924 |
| 14 | 0.334482 | 2.114079 |
| 15 | 0.094732 | 8.620485 |
| 16 | 0.075363 | 3.107303 |
| 17 | 0.002254 | 98.71131 |
| 18 | 0.235647 | 10.35458 |
| 19 | 0.336808 | 5.86112 |
| 20 | 0.169917 | 3.59219 |
| 21 | 0.130584 | 4.012811 |
| 22 | 0.582367 | 1.730911 |
| 23 | 0.214003 | 0.385673 |
| 24 | 0.0937 | 1.194442 |
