# Supplementary material for: Pim-1 kinase is a target of miR-486-5p and eukaryotic translation initiation factor 4E, and plays a critical role in lung cancer
Source: Mol Cancer. 2014 Oct 24;13:240. doi: 10.1186/1476-4598-13-240 (PMC4213487; doi:10.1186/1476-4598-13-240)

### Additional file 2 – Supplementary FigureS1:

**The effects of si-Pim-1 on cell proliferation in A549 cell in vitro.** A549 cells were transfected with Pim-1 siRNA at a final concentration of 50nM. Cell proliferation was determined by clone formation assay. 500 cells were replated in six well plates after 24-hour posttransfection. After ten days culture, visible colonies were fixed with methanol, stained with crystal violet. The experiments were performed at least three times.


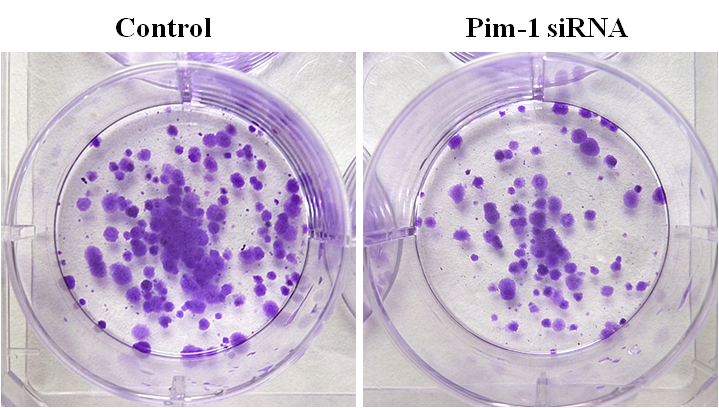

Supplement: Supplementary file 2 — Additional file 2: Figure S1: The effects of si-Pim-1 on cell proliferation in A549 cell in vitro. A549 cells were transfected with Pim-1 siRNA at a final concentration of 50nM. Cell proliferation was determined by clone formation assay. 500 cells were replated in six well plates after 24-hour posttransfection. After ten days culture, visible colonies were fixed with methanol, stained with crystal violet. The experiments were performed at least three times. (DOCX 674 KB) [file 12943_2014_1440_MOESM2_ESM.docx]
