# Supplementary material for: Pim-1 kinase is a target of miR-486-5p and eukaryotic translation initiation factor 4E, and plays a critical role in lung cancer
Source: Mol Cancer. 2014 Oct 24;13:240. doi: 10.1186/1476-4598-13-240 (PMC4213487; doi:10.1186/1476-4598-13-240)

**Additional file3 – Supplementary Figure S2:**

**Pim-1 protein expressions were overexpressed in NSCLC cell lines (A549, H1299, H157, SK-MES-1 and H358) compared to the average level of Pim-1 in normal lung tissues by Western Blot assay.** The level of Pim-1 protein in 24 cases of human normal lung tissue was used as negative control, and the relatively expression of Pim-1 protein in cell lines was normalized to the normal lung tissues.


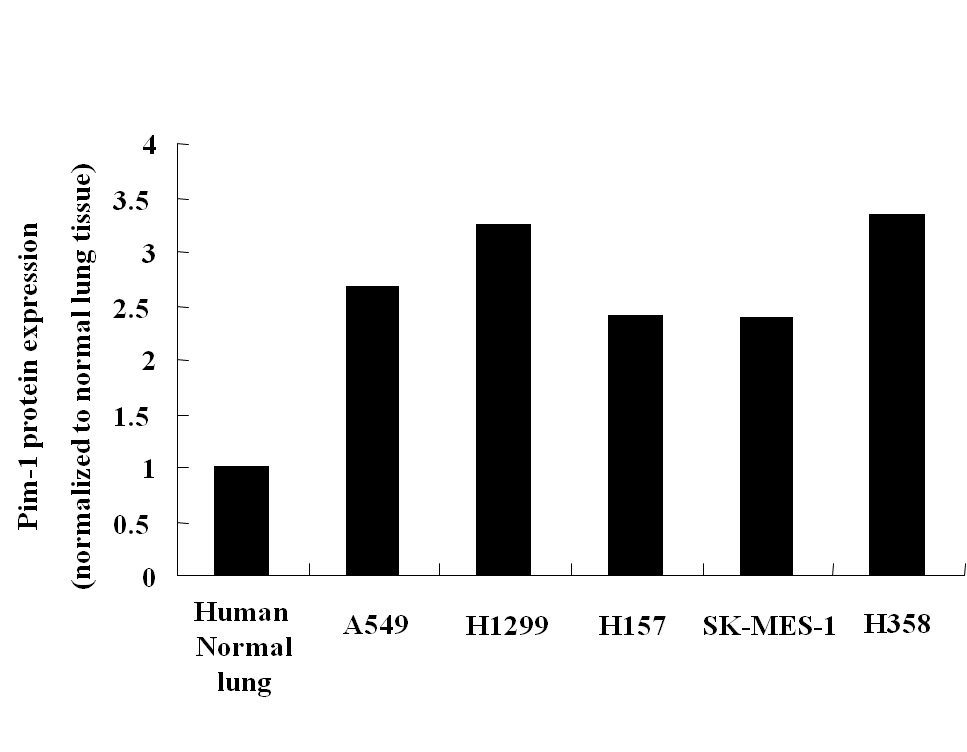

Supplement: Supplementary file 3 — Additional file 3: Figure S2: Pim-1 protein expressions were overexpressed in NSCLC cell lines (A549, H1299, H157, SK-MES-1 and H358) compared to the average level of Pim-1 in normal lung tissues by Western Blot assay. The level of Pim-1 protein in 24 cases of human normal lung tissue was used as negative control, and the relatively expression of Pim-1 protein in cell lines was normalized to the normal lung tissues. (DOCX 90 KB) [file 12943_2014_1440_MOESM3_ESM.docx]
