# Supplementary material for: Pim-1 kinase is a target of miR-486-5p and eukaryotic translation initiation factor 4E, and plays a critical role in lung cancer
Source: Mol Cancer. 2014 Oct 24;13:240. doi: 10.1186/1476-4598-13-240 (PMC4213487; doi:10.1186/1476-4598-13-240)
Supplement: Supplementary file 4 — Additional file 4: Figure S3: Pim-1 Knockdown increased the sensitivity of H1299 cells to gefitinib and cisplatin. H1299 cells were transfected with negtive control or Pim-1 siRNA for 24 h and then exposed to 20 μM gefitinib for 24 h (A) or 20 μM cisplatin for 48 h (B) respectively. After the aforementioned treatments, cell viability was assessed by MTT assays. All data were obtained from three independent experiments and shown as mean ± s.d. #P < 0.05 compared with each treatment alone. (DOCX 59 KB) [file 12943_2014_1440_MOESM4_ESM.docx]

**Additional file 4 – Supplementary Figure S3:**

**Pim-1 Knockdown increased the sensitivity of H1299 cells to gefitinib and cisplatin.** H1299 cells were transfected with negtive control or Pim-1 siRNA for 24h and then exposed to 20 μM gefitinib for 24h (A) or 20μM cisplatin for 48h (B) respectively. After the aforementioned treatments, cell viability was assessed by MTT assays. All data were obtained from three independent experiments and shown as mean ± s.d. #P<0.05 compared with each treatment alone.


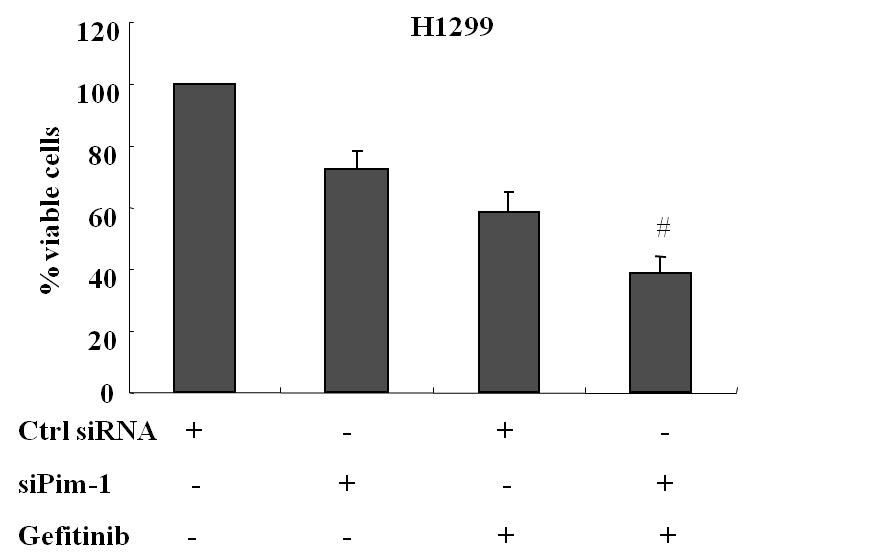


**A**


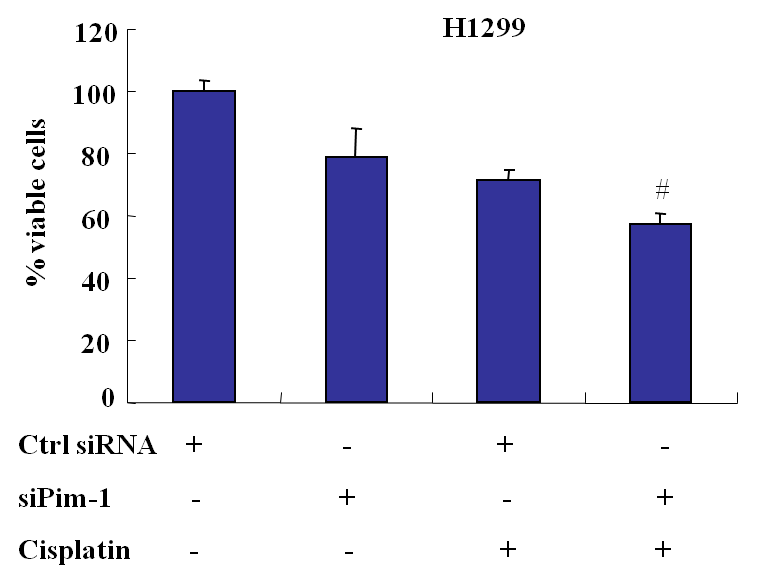


**B**
